# Efficacy of Herbivore-Induced Plant Volatile Methyl Salicylate in Evaluating the Seasonal Abundance of Herbivorous Thrips (Thysanoptera: Thripidae) in Sweet Pepper

**DOI:** 10.3390/insects15030156

**Published:** 2024-02-25

**Authors:** Uzoamaka C. Abana, Kaushalya G. Amarasekare

**Affiliations:** Department of Agricultural Sciences and Engineering, College of Agriculture, Tennessee State University, 3500, John A. Merritt Blvd., Nashville, TN 37209, USA; uzoamaka.abana@gmail.com

**Keywords:** *Frankliniella tritici*, methyl salicylate, neryl (S)-2-methylbutanoate, biological control, integrated pest management (IPM), seasonal abundance

## Abstract

**Simple Summary:**

Pest thrips species damage fruits, vegetables, field crops, and ornamental plants. They are difficult to control effectively using only insecticides. Semiochemicals produced by plants or animals are useful in integrated pest management (IPM) to reduce insecticide use in crop production. These chemicals attract arthropod pests and/or natural enemies of arthropod pests into cropping systems. Herbivore-induced plant volatiles (HIPV) or plant-produced semiochemicals such as methyl salicylate (MS) attract natural enemies and can be used as a pest management tactic. This research, conducted in Tennessee in 2018 and 2019, focused on investigating the efficacy of MS and the aggregation pheromone neryl (S)-2-methylbutanoate (NMB), an attractant of western flower thrips *Frankliniella occidentalis*, a severe pest of many crops, as a thrips management technique when compared with a no-lure control using sweet pepper as the crop plant. Our results show that the key species of thrips attracted to both lure treatments and the no-lure control was *Frankliniella* *tritici*, a significant pest of young cotton plants, which can also reduce the pest pressure from *F. occidentalis* by increasing its numbers in crop plants, banker plants, or trap crops. We discuss the possibility of using MS lure as a herbivorous thrips management practice in crop production.

**Abstract:**

Herbivorous thrips that damage fruits, vegetables, field crops, and ornamentals are challenging to control using insecticides and need an integrated approach (IPM) for their management. Herbivore-induced plant volatiles (HIPVs) are semiochemical plants produced to attract natural enemies (NEs) of arthropod herbivores. Sex pheromones are animal-based semiochemicals that can attract males or females of conspecifics. The HIPV methyl salicylate (MS) is used in IPM to attract NEs. We conducted field experiments in 2018–2019 in Tennessee to study the efficacy of MS and the aggregation pheromone neryl (S)-2-methylbutanoate (NMB), which attracts *Frankliniella occidentalis* (FO), a dominant pest of many crops, in attracting thrips using sweet peppers. We found a significantly higher number of thrips in traps baited with MS than in the traps containing NMB when compared with a no-lure control. All treatments caught only one thrips species, *Frankliniella tritici* (FT), a significant pest of young cotton. It can also lower the abundance of FO in other crops. Our findings show that although FO was not found in the study location in Tennessee, traps baited with MS are suitable for managing FT and reducing FO in susceptible crops by increasing FT and attracting NEs to crop productions that use IPM-based management practices.

## 1. Introduction

Herbivorous thrips can inflict injuries on a wide variety of crops, including fruits, vegetables, field crops such as cotton, and ornamental plants [1,2,3,4,5,6]. Most of these thrips species are significant pests of many vegetable crops, including peppers (*Capsicum annum* L.) [1,2,3,4,5,6,7]. In the United States, thrips species like western flower thrips *Frankliniella occidentalis* (Pergande), melon thrips *Thrips palmi* Karny, and the flower thrips *Frankliniella tritici* (Fitch) damage or are found on vegetable crops like sweet peppers [7,8,9].

Semiochemicals such as herbivore-induced plant volatiles (HIPVs) and pheromones are critical in the integrated management (IPM) of vegetable pests [10]. They are chemical compounds released by plants or animals and transmit signals between individuals that elicit behavioral, physiological, and biochemical changes in the second recipient [10]. Animal-based semiochemicals such as pheromones transmit signals between conspecifics, while allelochemicals modify the behavior of different species [10,11]. These attributes make plant and animal semiochemicals effective agents for pest management [12].

Plants use HIPVs as a defensive mechanism against chewing and sucking herbivorous arthropods [13]. Some plants respond to chemicals in the herbivores’ saliva by releasing HIPV [14,15,16]. Plants infested with herbivores emit higher HIPV quantities than non-infested plants, which tend to attract arthropod predators and parasitoids [17,18,19]. Most arthropod predators and parasitoids rely on HIPVs to detect herbivore-infested plants [20]. Apart from attracting natural enemies, some HIPVs attract herbivores [21,22].

Thrips feeding on crops such as sweet peppers reduce market quality due to the aesthetic damage caused by the pest [23]. Thrips management is, therefore, an important economic priority for many crops. Previous studies have shown that some HIPVs and pheromones attract thrips and arthropod predators in fruit crops. Methyl salicylate (MS) is a common HIPV used in agricultural systems to attract natural enemies to crop fields [21,22,24]. Gadino et al. [21] found that MS lures to attract predatory mites and other natural enemies also attracted thrips, a pest of grapes. Lee [22] also found that MS lures attracted thrips on strawberries. According to Salamanca et al. [24], herbivorous thrips were attracted to MS on cranberry. According to Lee [25], MS attracted many thrips to sticky traps placed on ornamental plants during the first year of the study. None of these studies specified the species of thrips that MS attracts on the respective fruit crops or ornamental plants. Information on species of thrips attracted to MS on vegetables is also limited. In addition to HIPVs, animal-based semiochemicals such as insect pheromones can act as aggregation or sex pheromones [12,26]. Insects also use these pheromones to protect themselves from predators and mass attack hosts to overcome host resistance [12].

The semiochemical neryl (S)-2-methylbutanoate (NMB) is an aggregation pheromone produced by adult males of one of the most damaging pest thrips species, the western flower thrips *Frankliniella occidentalis* (Pergande) [26,27]. It attracts both males and females of *F*. *occidentalis* [26]. Lures with NMB also attract predatory insects such as *Orius insidiosus* (Say), the most important predators of thrips. Neryl (S)-2-methylbutanoate guides the foraging habits of *O*. *insidiosus* and attracts both males and females of this species [28,29].

This study focused on determining the efficacy of laboratory-prepared lure of the HIPV methyl salicylate (MS) and a commercially available *F*. *occidentalis* aggregation pheromone neryl (S)-2-methylbutanoate (NMB) lure in attracting herbivorous thrips and the natural enemy *O*. *insidiosus* and their seasonal abundance on sweet pepper.

## 2. Materials and Methods

### 2.1. Field Site Selection and Study Setup

We conducted field experiments during the summer and fall of 2018 and 2019 at the Tennessee State University (TSU) Agricultural Research and Education Center (AREC) in Nashville, TN, USA. To obtain sweet pepper transplants for the field planting, we used 50-cell plastic planting trays (T. O. Plastics, Canby, OR, USA) with a moisture control potting mix (MiracleGro^®^) (Miracle-Gro Lawn Products, Inc., Marysville, OH, USA) for sowing the sweet pepper seeds (*C*. *annum* L. var. ‘California Wonder’) (W. Atlee Burpee Co., Warminster, PA, USA) in the greenhouse at the AREC on 23 April 2018 and 27 May 2019, respectively. We watered the trays on a needed basis, from seed sowing to transplanting.

We prepared the selected field site and arranged the field plots using a randomized complete block design (RCBD) with four and three blocks in 2018 and 2019, respectively. Due to some technical issues in the greenhouse that were beyond our control, we could not obtain the required number of healthy seedlings for four blocks in 2019. When the pepper plants were approximately 7 wk old, we transplanted them manually on 12 June 2018 and 15 July 2019, respectively. These technical issues in the greenhouse in 2019 caused a delay in obtaining and transplanting the seedlings in mid-June 2019. We grew the sweet pepper plants in raised beds using standard horticultural practices. Each bed (treatment plot within a block) (3 m × 3 m) was of five linear rows with ten plants per row per treatment. Plant-to-plant spacing within a row and between rows was 0.3 m and 0.6 m, respectively. Spacing for plot to plot within a block and block-to-block was 3 m and 5 m, respectively. We applied Ultrasol 20-20-20 water-soluble fertilizer (SQM, Atlanta, GA, USA), manually weeded the plots as often as needed, and conducted the experiments without insecticide applications throughout the study.

### 2.2. Treatments

The treatments were (i) laboratory-prepared lures of a HIPV (methyl salicylate [MS]), (ii) commercially purchased lures of an aggregation pheromone of *Frankliniella occidentalis* (neryl (S)-2-methylbutanoate [NMB]), and (iii) a no-lure control. We used commercially purchased liquid MS (Sigma-Aldrich, St. Louis, MO, USA) to prepare the lures in the laboratory. We made each MS lure using 6-mil polyvinyl tubing (Associated Bag Company, Milwaukee, WI, USA) and a cotton wick (large, braided cotton rolls) (Richmond Dental, Charlotte, NC, USA). After heat-sealing one end of the polyvinyl tubing, we placed a cotton wick inside the tubing and dispensed 3.5 mL of MS onto the cotton wick using a pipette. We heat-sealed the opening end of the polyvinyl tubing, placed them in aluminum foil bags (Associated Bag Company, Milwaukee, WI, USA), heat-sealed the bag opening, and stored them in a freezer until use. We adopted the methods used by Jones et al. [30] and Amarasekare and Shearer [31] to prepare the MS lures. We used commercially purchased NMB lures (ThriPher^®^, Biobest Biological Systems Ltd., Leamington, ON, Canada) in 2018 and 2019.

We attached each lure (either MS or NMB) to a white delta trap (Alpha Scents Inc., West Linn, OR, USA) using a metal wire and placed one-half (23 cm by 13.5 cm) of a back-folded yellow sticky card (Alpha Scents Inc., West Linn, OR, USA) on the interior bottom of the delta trap. We also utilized a delta trap and a sticky card for the no-lure control. We used white delta traps for this study because they were the only commercially produced neutral-color delta traps available to purchase at the time of the study. The color of the sticky cards did not play a significant role as a visual clue because the sticky cards were placed on the bottom of the trap and were not visible to the outside. We randomly assigned the three treatments to study plots within each block and hung each delta trap with a lure and a sticky card on a cross-shaped and 1.8 m tall PVC structure (custom-made) erected at the center of each experimental plot before hanging the traps. Traps were hung approximately 76 cm above the ground on 9 July 2018 and 10 July 2019, respectively, for the 2018 and 2019 experiments. We replaced the sticky cards and lures at weekly and monthly intervals, respectively. Preliminary studies show that these lures are effective for 4–5 wk. According to Gadino et al. [21] and Rodriguez-Saona et al. [32], replacing the whole lure or adding the MS to an existing lure increases its efficiency. We labeled the collected sticky cards, covered each with plastic wrap, and stored them in a freezer until checked.

### 2.3. Yield Data Collection

When fruits started to mature, we collected the yield from each plot, thoroughly checked each fruit for thrips’ damage, weighed both damaged and undamaged yields, and recorded them. We could not collect the yield data in 2019 due to the long-term drought experienced from July to October that directly affected plant growth and yield.

### 2.4. Statistical Analyses

We performed a two-way repeated measure analysis of variance (ANOVA) to find the significance of the main effects (year treatment and date) and their interaction (Proc Mixed) [33] and a one-way analysis of variance (ANOVA) for the yield data to find the significance among treatments. We compared the means of thrips and *O*. *insidiosus* individuals collected from traps at the *p* ≤ 0.05 significance level and the means of undamaged and damaged yield at the *p* ≤ 0.1 significance level (least square means [LSMEANS]) [33].

### 2.5. Thrips and Orius insidiosus Identification

Thrips and *Orius insidiosus* samples collected from the yellow sticky cards using CitriSolv^TM^ solution (VWR, Suwanee, GA, USA) were stored in 70% ethyl alcohol in glass vials and sent for identification. Cheryle A. O’Donnell, Systematic Entomology Laboratory, Agriculture Research Service, United States Department of Agriculture (SEL-USDA-ARS) identified the thrips individuals to species level. According to Thomas J. Henry (SEL-USDA-ARS), *O*. *insidiosus* is the only *Orius* spp. found in Tennessee.

### 2.6. Voucher Specimens

We deposited thrips and *O*. *insidiosus* voucher specimens in the insect collection at TSU’s Department of Agricultural and Environmental Sciences, Fruit, Vegetable, and Field Crop Entomology Laboratory in Nashville, TN, USA.

## 3. Results

### 3.1. Thrips 2018 and 2019

Thrips were the most abundant insect species captured in all treatments in 2018 and 2019. They were identified as flower thrips (*F*. *tritici*) with assistance from the SEL-USDA-ARS.

There was a significant interaction between year × treatments (F = 93.05, df = 2, 181, *p* < 0.0001), year × date (F = 9.25, df = 7, 181, *p* < 0.0001), and year × treatment × date (F = 6.34, df = 48, 181, *p* < 0.0001) for the weekly mean number of *F*. *tritici* trapped in sticky cards. Thus, we analyzed the data for each year using a repeated measure ANOVA (Proc Mixed) [33]. There was a significant interaction between treatment and date for 2018 (F = 8.01, df = 24, 107, *p* < 0.0001) and 2019 (F = 3.46, df = 24, 72, *p* < 0.0001) for the mean number of *F*. *tritici* trapped in the sticky cards. These interactions may be due to the drastic increase and decrease in *F*. *tritici* populations throughout the study period in 2018 and 2019. We analyzed the data for each date as simple means using a one-way ANOVA (Proc Mixed) and compared the treatment means using LSMEANS in 2018 and 2019 [33] (Table 1 and Table 2).

#### 3.1.1. 2018

**Figure 1:**
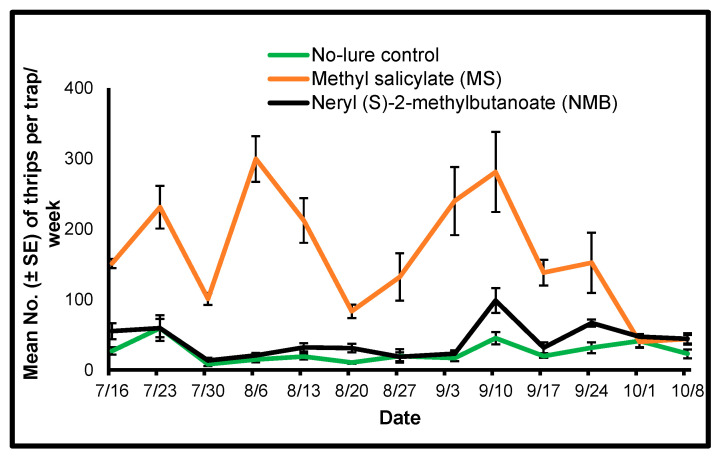
Mean (±SE) number of *Frankliniella tritici* per Delta trap per week counted from yellow sticky cards collected from traps placed in sweet pepper plots using two semiochemical lures (1: herbivore-induced plant volatile [HIPV] MS [methyl salicylate]; 2: thrips aggregation-pheromone NMB [neryl (S)-2-methylbutanoate]) and a no-lure control during 2018 in Nashville, Tennessee.

Our results show a significant treatment effect for each trap collection day in July, August, and September 2018 (Table 1). In addition, significantly higher mean numbers of *F*. *tritici* were trapped in the traps with methyl salicylate (MS) lure on each trap collection day from July to September when compared with the mean number of *F*. *tritici* trapped in the traps with the neryl (S)-2-methylbutanoate (NMB) lure or no-lure control on the respective trap collection days (Table 1 and Figure 1). We counted a significantly higher number of *F*. *tritici* from traps with the NMB lure on the weekly collection dates of 16 July, 20 August, and 17 September 2018 when compared with the mean number of *F*. *tritici* trapped on the sticky cards of no-lure control on the respective trap collection days (Table 1). *Frankliniella tritici* was abundant in the range of ~50–300 throughout the trapping period (Figure 1).

#### 3.1.2. *Orius insidiosus*

**Figure 2:**
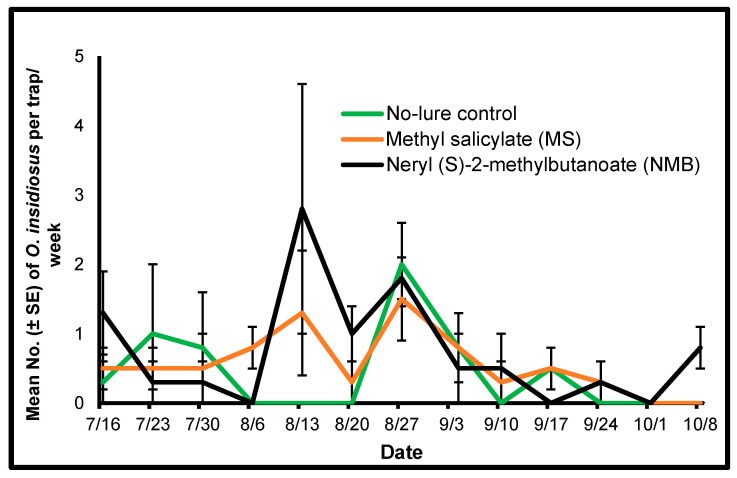
Mean (±SE) number of *Orius insidiosus* per Delta trap per week counted from yellow sticky cards collected from traps placed in sweet pepper plots using two semiochemical lures (1: herbivore-induced plant volatile [HIPV] MS [methyl salicylate]; 2: thrips aggregation-pheromone NMB [neryl (S)-2-methylbutanoate]) and a no-lure control during July to October 2018, in Nashville, Tennessee.

There was a significant interaction between treatment and the date of weekly trap collection in attracting *O*. *insidiosus* to each treatment (F = 8.01, df = 24, 107, *p* ≤ 0.0001). These interactions may be due to the population fluctuations of this species that occurred throughout the study period. We analyzed the data for each date as simple means using a one-way ANOVA (Proc Mixed) and compared the treatment means using LSMEANS. There was a significant treatment effect for the mean number of *O*. *insidiosus* attracted to the treatments on 6 August and 8 October 2018 (F = 9.0, df = 2, 6, *p* = 0.01 and F = 9.0, df = 2, 6, *p* = 0.01, respectively). There were significantly higher mean numbers of this species caught in the methyl salicylate treatment compared with the mean number caught in the no-lure control (t = −3.67, df = 6, *p* = 0.01) or the neryl (S)-2-methylbutanoate treatment (t = 3.67, df = 6, *p* = 0.01) (LSMEANS) on 6 August. There were significantly higher mean numbers of *O*. *insidiosus* caught in the neryl (S)-2-methylbutanoate treatment when compared with the mean number caught in the no-lure control (t = −3.67, df = 6, *p* = 0.01) or the methyl salicylate treatment (t = −3.67, df = 6, *p* = 0.01) (LSMEANS) on 8 October. *Orius insidiosus* was not abundant in high numbers throughout the study (Figure 2).

### 3.2. Yield Data

The mean weight (±SE) of undamaged sweet pepper fruits obtained from methyl salicylate, neryl (S)-2-methylbutanoate, and no-lure control treatments were 40.7 ± 12.0, 33.2 ± 4.5, and 26.3 ± 5.4 kg, respectively. There was no significant treatment effect for the weight of the undamaged fruits (F = 2.02, df = 2, 6, *p* = 0.21). Although statistically insignificant, we obtained the highest yield from the MS treatment. The mean weight of damaged sweet pepper fruits obtained from MS, NMB, and no-lure control treatments were 2.0 ± 0.8, 5.3 ± 0.2, and 12.4 ± 3.5 kg, respectively. There was a significant treatment effect for the weight of the damaged fruits (F = 6.43, df = 2, 6, *p* = 0.03). The damaged yield of sweet pepper fruits was significantly higher for the fruits collected from the no-lure control when compared with the damaged yield collected from the MS (t = 3.52, df = 6, *p* = 0.01) or NMB treatment (t = 2.38, df = 6, *p* = 0.06) at *p* ≤ 0.1 significance level. There was no significant difference between the damaged yield collected from the MS and NMB treatments.

### 3.3. 2019

#### 3.3.1. Thrips

**Figure 3:**
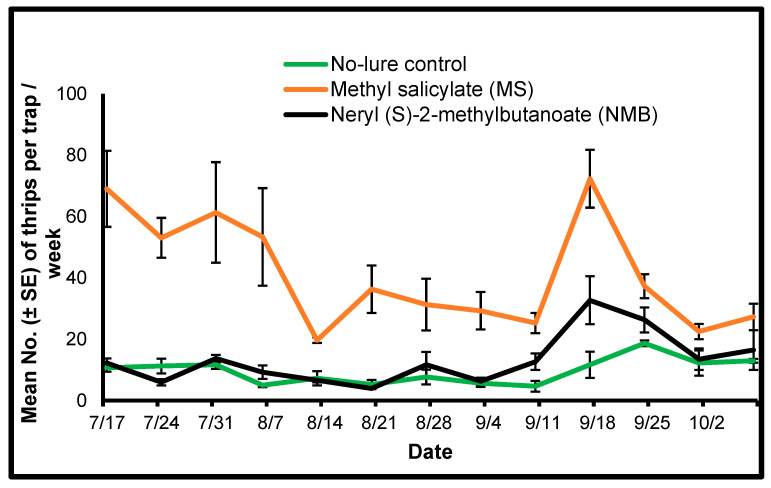
Mean (±SE) number of *Frankliniella tritici* per Delta trap per week counted from yellow sticky cards collected from traps placed in sweet pepper plots using two semiochemical lures (1: herbivore-induced plant volatile [HIPV] MS [methyl salicylate]; 2: thrips aggregation-pheromone NMB [neryl (S)-2-methylbutanoate]) and a no-lure control during 2019 in Nashville, Tennessee.

All the collected thrips individuals were identified as *F*. *tritici*. Similar to 2018, there was a significant treatment effect for the mean number of *F*. *tritici* caught on each trapping day in July, August, and September 2019 (Table 2). A significantly higher number of *F*. *tritici* were caught in the traps with MS lure when compared with the mean number of *F*. *tritici* in traps with NMB lure or no-lure control in July, August, and September 2019 (Figure 3). Except for 17 September 2019, there was no significant difference between the mean numbers of *F*. *tritici* caught in the traps with NMB lure when compared with the mean number of *F*. *tritici* caught in the traps with no-lure control (Table 2). *Frankliniella tritici* was abundant in the MS lure traps in the range of 20–70 throughout the trapping period from July to October (Figure 3).

#### 3.3.2. *Orius insidiosus*

The mean number of *O*. *insidiosus* trapped in all three treatments was negligible in 2019. Because of this reason, we did not perform any statistical analyses for the *O*. *insidiosus* data.

## 4. Discussion

Semiochemicals from plants and/or arthropods that attract herbivorous arthropods and their natural enemies are environmentally safe alternatives to pesticide use [21,22]. Improper or overuse of pesticides can pollute land and water resources and negatively impact human health, livestock, wildlife, fish, and other aquatic animals [34]. Previous studies have shown that semiochemicals derived from plants, such as herbivore-induced plant volatiles (HIPVs), attract natural enemies to pest-density areas [12]. Some studies show that certain HIPVs can attract arthropod pest species and their natural enemies [21,22,24]. Here, we investigated the relative efficacy of two semiochemical lures, the HIPV methyl salicylate (MS) and thrips aggregation-pheromone neryl (S)-2-methylbutanoate (NMB) in attracting thrips and their predator (*O*. *insidiosus*) using sweet pepper as the crop plant. Results from our two years of field experiments show that MS attracts flower thrips *F*. *tritici* and *O*. *insidiosus* in sweet peppers.

Using HIPVs to trap pest arthropods or attract their natural enemies to crop fields is an environmentally sustainable pest management practice [30]. Our findings suggest that this technique may provide a cost-effective and environmentally safe means of pest management. Indeed, we found consistent trends in attracting many *F*. *tritici* to MS lures. Traps with MS lure caught a significantly higher number of *F*. *tritici* than the traps with NMB lures compared with a no-lure control. Other common thrips species, such as *F*. *occidentalis* and *T. palmi*, were not found in any of the traps in all three treatments in this study, even though the NMB lure is specifically targeted to trap the males and females of *F*. *occidentalis*. We did not find any *F*. *occidentalis* in pepper flowers collected from a separate study conducted without semiochemical lures in 2018, which was aimed at finding *O*. *insidiosus* and thrips abundance in sweet peppers when using ornamental peppers as banker plants [35].

The number of *O*. *insidiosus* was low throughout the study period in 2018 and 2019. We speculate that this low population of the Anthocorid bug was due to the high summer and early fall temperatures in Tennessee. The suitable temperature range for *O*. *insidiosus* development is between 19 °C and 31 °C, with the optimum temperature at 25 °C [36]. The monthly average maximum temperatures for June, July, and August in 2018 and June, July, August, and September 2019 were above 32 °C [37]. The days above a 31 °C maximum temperature in June, July, August, and September were 20, 26, 23, and 16 in 2018 and 16, 26, 26, and 27 in 2019 [37]. Although we did not collect pepper flowers in this study, *O*. *insidiosus* and thrips individuals are more commonly found on flowers than in outside environments, which can provide floral resources as food sources and refugia for both insects when environmental conditions are harsh (Amarasekare personal observation).

According to Baez et al. [1], in a study on peppers conducted in Florida, 81% and 3% of the thrips individuals found on peppers in the fall were *F*. *tritici* and *F*. *occidentalis*, respectively. The lower abundance of *F*. *occidentalis* in the fall in Florida could also be why we did not trap *F*. *occidentalis* in our studies in Tennessee in the early fall of 2018 and 2019. We need adequate data to verify the presence and abundance of *F*. *occidentalis* in Tennessee. Field surveys conducted in 1996–1999 and 2009–2010 found no evidence of *F*. *occidentalis* in Tennessee during the study period [5,38]. Our study confirms these previous findings and raises the critical question of the presence of *F*. *occidentalis* in Tennessee, specifically during summer and early fall.

Flower thrips are common herbivorous thrips species that can damage cotton seedlings [3,39]. *Frankliniella tritici* has been reported to infest cotton seedlings in 10 U.S. states, while *F*. *occidentalis* has been observed infesting cotton seedlings in 13 U.S. states [3], and both species are controlled by insecticides. A study conducted in Florida showed that 98% of the adult thrips found in cotton flowers were *F*. *tritici* when compared with *F*. *occidentalis* and other thrips species [39]. Results from a study to evaluate the interspecific competition between *F*. *tritici* and *F*. *occidentalis* using the crop *C*. *annuum* and the weed species *Raphanus raphanistrum* L. show that *F*. *tritici* is competitively superior to the invasive *F*. *occidentalis* and *F*. *tritici* could cause the competitive exclusion of *F*. *occidentalis* when they coexist. [40]. This information shows that methyl salicylate lures could be useful in trapping *F*. *tritici* in cotton fields and attracting it to other crop fields where *F*. *occidentalis* is a major pest.

This study shows that the semiochemical methyl salicylate could be utilized as an integrated pest management technique to manage flower thrips *F*. *tritici* and *F*. *occidentalis* in agricultural cropping systems. These lures could be incorporated into thrips management programs in organic and conventional crop production systems where *F*. *tritici* is an issue to trap and exclude this species or attract it to crop fields with high populations of *F*. *occidentalis* to compete and exclude the latter. The important broader implication of our results is that the use of lures of methyl salicylate, which is both more cost-effective and less labor-intensive crop management practice, benefits crop IPM programs in providing an environmentally safe alternative to reduce pesticide use in IPM.

## Figures and Tables

**Table 1:** Simple means analysis of variance (ANOVA) statistics for the mean number of *Frankliniella tritici* per trap per week collected from sweet pepper plots using two semiochemical lures (1: herbivore-induced plant volatile [HIPV] MS [methyl salicylate]; 2: thrips aggregation-pheromone NMB [neryl (S)-2-methylbutanoate]) and a no-lure control during 2018, in Nashville Tennessee.

Date Traps Collected				Treatment Comparison Statistics (LSMEANS)								
	Simple Means ANOVA			No-Lure Control and Methyl Salicylate (MS)			No-Lure Control and Neryl (S)-2-Methylbutanoate (NMB)			Neryl (S)-2-Methylbutanoate (NMB) and Methyl Salicylate (MS)		
	F	df	*p*	t	df	*p*	t	df	*p*	t	df	*p*
16 July	61.64	2, 6	<0.0001	−10.54	6	<0.0001	−2.39	6	0.05	8.20	6	0.0002
23 July	28.66	2, 6	0.001	−6.55	6	0.001	0.01	6	0.99	6.56	6	0.001
30 July	88.15	2, 6	<0.0001	−11.83	6	<0.0001	−0.70	6	0.51	11.13	6	<0.0001
6 August	73.44	2, 6	<0.0001	−10.61	6	<0.0001	−0.22	6	0.83	10.38	6	<0.0001
13 August	57.06	2, 4	0.0011	−9.94	4	0.001	−0.36	4	0.74	8.73	4	0.001
20 August	79.12	2, 6	<0.0001	−12.20	6	<0.0001	−3.44	6	0.014	8.76	6	0.0001
27 August	19.48	2, 5	0.0044	−5.53	5	0.001	0.06	5	0.96	5.58	5	0.003
4 September	29.59	2, 5	0.002	−6.92	5	0.001	−0.21	5	0.84	6.72	5	0.001
10 September	12.75	2, 6	0.007	−4.82	6	0.003	−1.09	6	0.32	3.72	6	0.01
17 September	21.21	2, 6	0.002	−6.48	6	0.001	−2.69	6	0.04	3.80	6	0.01
24 September	11.30	2, 5	0.014	−4.70	5	0.005	−1.51	5	0.19	3.32	5	0.02
1 October	0.68	2, 5	0.5444	0.40	5	0.702	−0.79	5	0.46	−1.12	5	0.31
8 October	3.04	2, 5	0.1370	−1.98	5	0.104	−2.21	5	0.08	−0.06	5	0.96

**Table 2:** Simple means analysis of variance (ANOVA) statistics for the mean number of *Frankliniella tritici* per trap per week collected from sweet pepper plots using two semiochemical lures (1: herbivore-induced plant volatile [HIPV] MS [methyl salicylate]; 2: thrips aggregation-pheromone NMB [neryl (S)-2-methylbutanoate]) and a no-lure control during 2019, in Nashville, Tennessee.

Date Traps Collected				Treatment Comparison Statistics (LSMEANS)								
	Simple Means ANOVA			No-Lure Control and Methyl Salicylate (MS)			No-Lure Control and Neryl (S)-2-Methylbutanoate (NMB)			Neryl (S)-2-Methylbutanoate (NMB) and Methyl Salicylate (MS)		
	F	df	*p*	t	df	*p*	t	df	*p*	t	df	*p*
17 July	20.95	2, 4	0.01	−5.69	4	<0.005	−0.16	4	0.88	5.52	4	0.01
24 July	62.89	2, 3	0.004	−9.33	3	0.003	1.58	3	0.213	9.65	3	0.002
31 July	9.60	2, 4	0.03	−3.87	4	0.02	−0.16	4	0.88	3.74	4	0.02
6 August	8.61	2, 4	0.04	−3.75	4	0.02	−0.34	4	0.75	3.41	4	0.03
13 August	24.12	2, 4	0.01	−5.85	4	0.004	0.32	4	0.77	6.17	4	0.004
20 August	17.84	2, 4	0.01	−5.06	4	0.01	0.22	4	0.83	5.28	4	0.01
27 August	9.30	2, 4	0.03	−4.03	4	0.02	−0.68	4	0.53	3.35	4	0.03
3 September	13.73	2, 4	0.02	−4.60	4	0.01	−0.13	4	0.90	4.47	4	0.01
10 September	15.27	2, 4	0.01	−5.48	4	0.01	−2.12	4	0.10	3.36	4	0.03
17 September	95.53	2, 4	0.0004	−13.47	4	0.0002	−4.66	4	0.01	8.80	4	0.001
24 September	8.00	2, 4	0.04	−3.98	4	0.02	−1.63	4	0.18	2.35	4	0.08
1 October	2.01	2, 2	0.33	−1.91	2	0.20	−0.22	2	0.85	1.55	2	0.26
8 October	4.12	2, 3	0.14	−2.78	3	0.07	−0.61	3	0.59	1.88	3	0.17

## Data Availability

All data is provided in the manuscript.
